# Effectiveness of Virtual Reality Intervention in Enhancing Motor and Cognitive Functions in Down Syndrome: A Systematic Review

**DOI:** 10.7759/cureus.80148

**Published:** 2025-03-06

**Authors:** Maheshkumar Baladaniya, Shraddha Baldania

**Affiliations:** 1 Department of Physical Therapy, Neighborhood Physical Therapy PC, New York, USA; 2 Department of Physical Therapy, Enjoy Rehab PT PC, New York, USA

**Keywords:** cognitive rehabilitation, down syndrome, physical rehabilitation, telerehabilitation, virtual reality

## Abstract

Down syndrome (DS) is a chromosomal disorder and is most commonly associated with cognitive impairments, motor dysfunction, and developmental delays. Various therapeutic interventions have been implemented to improve developmental milestones and enhance the quality of life in DS patients. Hence, this systematic review was performed to evaluate the impact of virtual reality (VR) based physical and cognitive rehabilitation in individuals with DS. The databases involved in the literature search consisted of PubMed, Science Direct, and Google Scholar. Studies were included if they assessed or evaluated VR-based therapeutic interventions in individuals with DS of any age. Moreover, randomized controlled trials (RCTs) with full-text availability in English were incorporated. Furthermore, a mixed-method appraisal tool was utilized to assess the quality of the studies. A total of seven RCTs that met the inclusion criteria were included. The included studies demonstrated that VR-based interventions significantly improved motor function, postural control, physical fitness, and independent skills. However, compliance with clinical guidelines and healthcare transition planning for DS patients remain areas of concern. Additionally, future research should focus on conducting large, high-quality RCTs with standardized assessment tools and long-term follow-ups to establish evidence-based guidelines associated with VR therapy for individuals with DS.

## Introduction and background

Down syndrome (DS) is a chromosomal abnormality that affects around one in every 675 births [1]. The features linked to DS mainly consist of small stature, facial dysmorphism, weak muscular tone, and joint hypermobility in addition to cognitive impairment. Additionally, there is a higher chance of developing autoimmune diseases, metabolic disorders, Type 1 diabetes, Alzheimer's disease, rheumatoid arthritis, celiac disease, congenital heart disease, childhood leukemia, sleep disorders, and developmental conditions like autism spectrum disorder [2]. Individuals with DS often experience developmental delays, cognitive impairments, and difficulties in motor function, which impact their overall quality of life [3,4]. Additionally, improvements in early identification and prenatal diagnosis have made it possible to provide appropriate medical care and early intervention [2,5,6].

Early execution of interventions (physical therapy, occupational therapy, speech therapy, and educational programs) plays a crucial role in improving functional independence and enhancing developmental outcomes in individuals with DS [7]. The goal of the physical therapy intervention is to maintain and enhance weight control, muscle strength, and cardiovascular capacity [8]. Additionally, physical therapy interventions have been reported to improve motor function, postural control, and balance, while speech and occupational therapy contribute to communication skills and activities of daily living [9-11]. Moreover, advancements in technology, such as virtual reality (VR)-based interventions and telerehabilitation approaches, have demonstrated promising results in enhancing motor and cognitive functions by offering an engaging, immersive, and interactive approach to therapy [12-14]. VR modalities are typically classified as non-immersive or immersive based on user immersion and presence. Non-immersive VR displays virtual environments on traditional two-dimensional (2D) screens. Users interact with these systems via a hand controller, a mouse, or a joystick [15]. Examples include Nintendo (Nintendo Company Limited, Kyoto, Japan) and Xbox (Microsoft Corporation, Redmond, WA) with Kinect Sensor. In contrast, immersive VR, less common in research and clinical settings, provides 360° full immersion via head-mounted displays [16]. The most widely used immersive VR devices in rehabilitation are Meta Quest 2 and 3 (Meta, Menlo Park, CA). Exergames are the main VR-based rehabilitation tools, especially for children, as their engaging nature boosts participation and enhances therapy outcomes [12]. VR-based interventions allow individuals with DS to practice motor skills, improve postural control, and enhance cognitive functions in a controlled, safe, and stimulating environment [12], thus emerging as a promising tool for improving cognitive [17-20] and motor functions in DS patients [12].

Compared to well-studied neurodevelopmental disabilities like cerebral palsy [21], limited literature is available on the use of VR for the neurorehabilitation approach for children and adolescents, potentially enhancing motor skills, balance, and physical fitness [22,13]. Previous reviews [14,23,24] demonstrated that VR outperforms conventional therapy in enhancing motor learning, balance, strength, and endurance. Although VR has been increasingly utilized in neurorehabilitation and motor training, its effectiveness specifically in DS evaluating motor and cognitive functions remains underexplored due to limited studies performed [17,25-30], highlighting the knowledge gap. Additionally, there is no systematic review (SR) reported assessing both the variables of motor and cognitive functions in DS patients. Hence, the growing interest [25-30] in VR applications for DS rehabilitation necessitates a systematic evaluation of existing evidence to determine its efficacy and clinical applicability. Therefore, the current systematic review aims to evaluate the effectiveness of VR-based interventions in improving motor skills, balance, and cognitive functions in DS patients.

## Review

Data sources and search strategy

For the execution of this SR, the Preferred Reporting Items for Systematic Reviews and Meta-Analyses (PRISMA) were followed [31]. The electronic databases along with the keywords (Medical Subject Headings) utilized consisted of ScienceDirect (“Down syndrome”, AND “Virtual Reality”, NOT “Augmented reality”), PubMed (“Down syndrome”, AND “Virtual Reality”, NOT “Augmented reality”), and Google Scholar (“Down syndrome”, AND “Virtual Reality”, NOT “Augmented reality”). The literature search was performed from January 2020 to January 2025. The therapeutic intervention part mainly involved postural balance, motor activity, motor skill, balance, gross motor function, motor performance, and cognitive function. The population, intervention, comparison, outcomes, and study design (PICOS) framework for the current review consisted of population = Down syndrome patients (children, adolescents, or adults), intervention = VR-based intervention in enhancing motor and cognitive functions, comparison = Conventional intervention (traditional physiotherapy, speech therapy, occupational therapy, motor skill training, cognitive training, or no intervention at all), outcomes = Motor performance, cognitive functions, balance (static and dynamic), postural control, functional independence, physical fitness, physical activity levels, and cognitive functions, and study design = Randomized controlled trials (RCTS), experimental studies, and observational studies.

Study screening and selection

Studies published between January 2020 and January 2025 consisting of individuals diagnosed with DS at any age, evaluating the impact of VR therapeutic intervention in the achievement of milestones in DS patients, and being observational studies (cohort studies, case-control studies) or RCTs with full-text availability in English were incorporated. Studies were excluded if they focused on prenatal diagnosis or specific medical conditions (e.g., congenital heart defects) commonly related to DS, were reviews, book chapters, or editorials, or reported inappropriate information related to the topic. For the removal of duplicates, blinded, independent screening of articles (titles and abstracts) was performed by both authors to discard evidence not following the criteria manually. Any discrepancies between reviewers in study selection were resolved through discussion, and as the consensus was achieved, the study was included. As a consensus was achieved mutually among both the reviewers, there was no requirement for the incorporation of the third reviewer.

Data extraction

The variables consisting of demographic characteristics of the studies (authors, year, design, sample size, age of individuals, and quality of studies) along with the summary of the inculcated studies (objectives, variables assessed, comparisons made, and statistical improvements shown by VR intervention) were extracted. The variables that were assessed consisted of study characteristics, type of intervention, exposure, comparator, or control intervention, and outcome measures associated with motor and cognitive functions (motor performance, cognitive functions, static and dynamic balance, postural control, functional independence, physical fitness, physical activity levels, and cognitive functions), and statistical key findings. The information was reviewed and merged in a tabular format.

Quality assessment

The Mixed Methods Appraisal Tool (MMAT) was employed for quality assessment of the included observational and RCTs [32]. Based on the various parameters described in the tool, the studies were graded as high (≥4 scores), low (≤1 score), or moderate quality (2-3 scores) [32]. For observational studies, the tool assessed the clarity of the research question, appropriateness of sampling strategy, adequacy of outcome measures, risk of bias (RoB) in data collection, and appropriateness of statistical analysis. Meanwhile, for RCTs, the tool assessed the randomization process, allocation concealment, blinding, completeness of outcome data, and adherence to intervention [32]. Moreover, the studies were also assessed using the Cochrane RoB 2.0 tool (https://www.riskofbias.info/welcome/robvis-visualization-tool) to assess the quality of RCTs [33].

Data synthesis

High-quality studies, along with their influencing factors, biases, and limitations were analyzed critically. The critical narrative technique was used which involves synthesis using text, tables, and figures, summarizing, and validating the outcomes of the evidences. The evidences incorporated showed a wide range of heterogeneity.

Results

**Figure 1:**
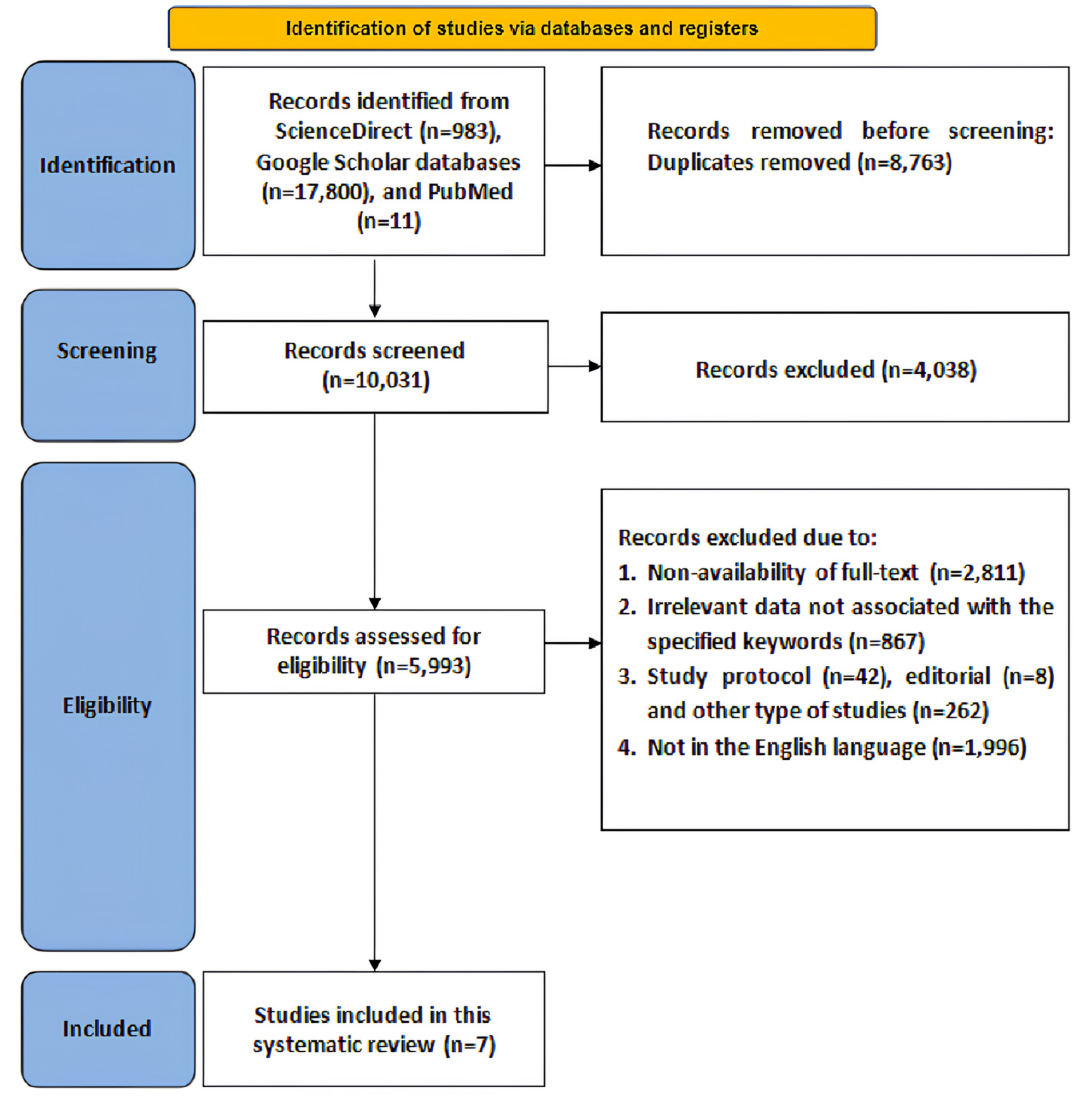
PRISMA flowchart illustrating search strategy PRISMA = Preferred Reporting Items for Systematic Reviews and Meta-Analyses

Overall, 10,031 articles were screened from PubMed, Science Direct, and Scopus databases. Figure 1 illustrates the selection strategy for the included studies. A total of 8,763 duplicate articles and 4,038 unretrieved were removed. Following this, 5,993 articles were screened for eligibility, out of which 2,811 lacked full-text availability, eight were editorials, 42 were study protocols, 262 were other study designs, 867 were irrelevant to the specified keywords, and 1,996 were not in English because of which these studies were excluded. Hence, in total, seven RCTs were inculcated.

**Figure 2:**
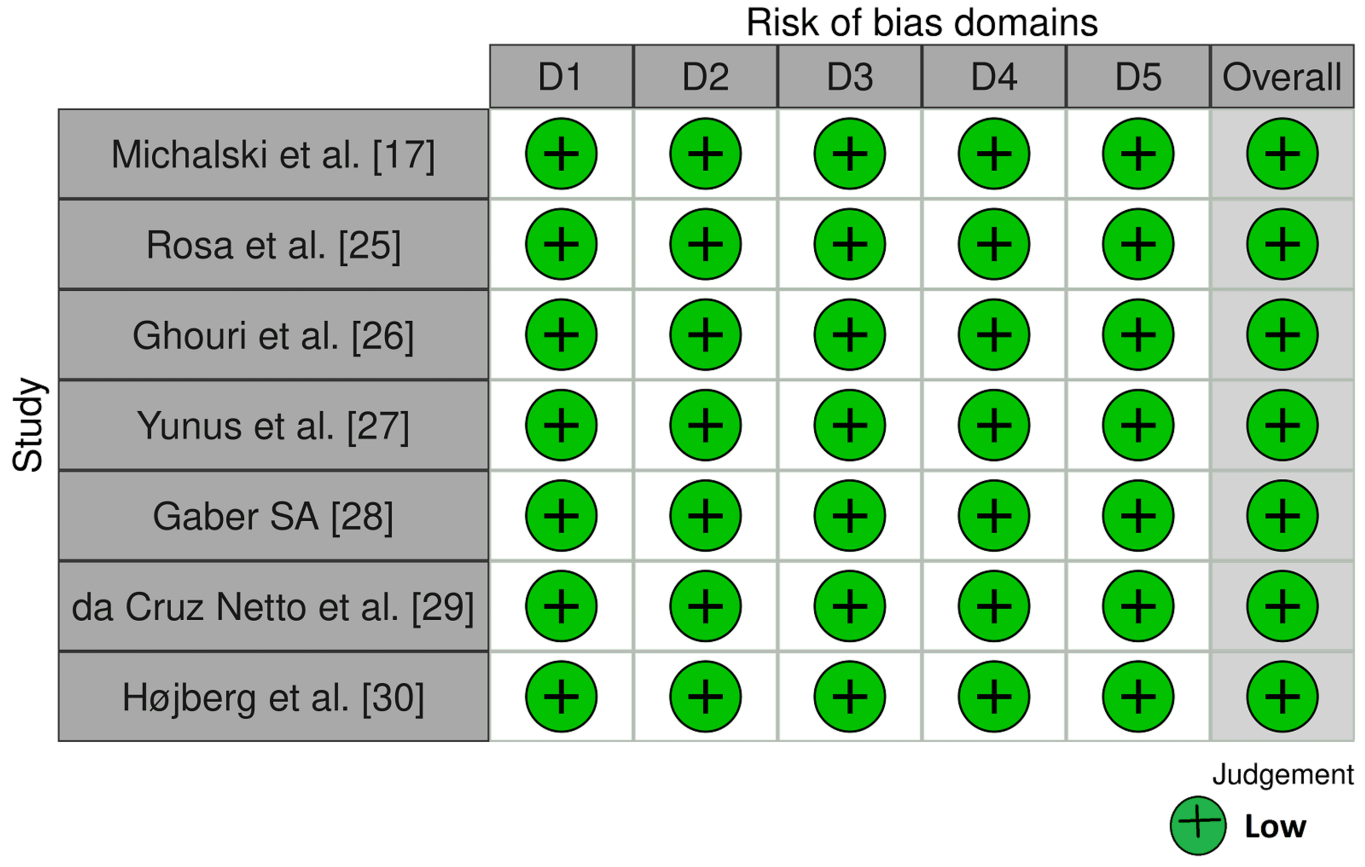
Traffic lights plot reporting low risk of bias in the included studies (RoB 2.0 tool) RoB = Risk of bias, D1 = Bias arising from the randomization process, D2 = Bias due to deviations from intended intervention, D3 = Bias due to missing outcome data, D4 =Bias in measurement of the outcome data, D5 = Bias in selection of the reported result

The demographic information along with the quality of the evidence (MMAT) is reported in Table 1. Moreover, the methodological quality of the RCTs using the RoB 2.0 tool is illustrated in Figure 2.

**Table 1:** Demographic characteristics of the reviewed studies DS = Down syndrome, TD = Typically developed

Sr. No.	Study	Year	Design	Sample size	Age of patients	Quality
1.	Michalski et al. [17]	2022	RCT	16	Mean age = 25.25±6.61 years	High
2.	Rosa et al. [25]	2023	RCT	68	DS mean age = 18.9±6.1 and with TD mean age = 19.0±1.6	High
3.	Ghouri et al. [26]	2024	RCT	24	6-9 years	High
4.	Yunus et al. [27]	2024	RCT	20	9-18 years	High
5.	Gaber SA [28]	2024	RCT	18	8-12 years	High
6.	da Cruz Netto et al. [29]	2020	RCT	30	10-22 years	High
7.	Højberg et al. [30]	2023	RCT	25	Mean age DS = 23.9 ± 3 years, TD = 22.8 ± 1.8 years	High

The objectives, methodological part, results, and conclusion of the evidence are demonstrated in Table 2.

**Table 2:** Summary of the reviewed studies describing impact of VR based interventions in DS patients DS = Down syndrome, VR = Virtual reality, VR SenMor = sensory-motor virtual reality, PA = Physical activity, RPE = Rate of Perceived Exertion, HR = Heart rate, TUG = Timed-up and go test, PBS = Pediatric balance scale, ISS = Independence Skills Scale, VATT = Visuomotor accuracy tracking task, TD = Typically developed.

Sr. No.	Author	Year	Objective	Variables assessed	Comparison	The statistical improvement shown by VR intervention
1.	Michalski et al. [17]	2022	To evaluate the feasibility and benefits of VR for learning.	Mood, attention, activity, impulses, anxiety, and withdrawal	Pre- and post-comparison within the groups.	Mean differences observed for mood (0.61±0.5), attention (0.66±0.4), activity (0.17±0.2), impulses (0.22±0.3), anxiety (0.19±0.3), and withdrawal (0.23±0.3). Both VR (P<0.001) and conventional (P=0.002) drawing significantly improved behavior, mood, and attention in a learning setting, with no significant difference between interventions (P=0.53).
2.	Rosa et al. [25]	2023	To assess the impact of VR on motor performance and PA.	HR, RPE, and functional performance	Pre-and post comparison in between the groups and within the groups.	HR (pre and post) = 94.1 ± 14.3 and 84.9 ± 12.6, respectively. RPE (pre and post) = 5.6 ± 2.1 and 3.7 ± 1.8, respectively. Performance through game score (pre and post) = 58.7 ± 12.5 and 78.3 ± 14.2 respectively.
3.	Ghouri et al. [26]	2024	To evaluate the impact of VR on balance (static and dynamic).	Static and dynamic balance	Pre- and post-comparison between the groups and within the groups (VR intervention and traditional intervention).	Mean PBS scores were 50.33±2.22 (VR intervention) and 49.41±3.20 (traditional intervention). Mean Rhomberg test scores were 29.08±1.16 (VR intervention) and 29.58±1.44 (traditional intervention) with eyes open. Whereas, for eyes closed the improvements were 28.17±2.40 (VR intervention) and 28±2.33 (traditional intervention).
4.	Yunus et al. [27]	2024	To assess the impact of VR SenMor on balance (static and dynamic).	Static balance (PBS), and dynamic balance (TUG)	Pre- and post-comparisons within the groups and in between the groups (VR and control).	In VR intervention, PBS (pre and post = 37.18 ± 9.87 and 43.18 ± 9.34. TUG (pre and post) = 12.57 ± 0.99 and 8.96 ± 0.91. In the control group, PBS (pre and post = 28.90 ± 7.64 and 29.10 ± 7.11. TUG (pre and post) = 13.48 ± 1.55 and 13.50 ± 1.62.
5.	Gaber SA [28]	2024	To enhance independence skills (ISS) in children with moderate DS using a VR training program.	Independent living skills	Pre- and post-comparison within the groups.	The VR group showed a significant improvement in ISS scores from pre- to post-measurement (z = -2.207, p = 0.027), as did the regular training group (z = -2.201, p = 0.028). No significant change was observed in the control group (z = -0.647, p = 0.518). Post-intervention, ISS scores significantly differed among groups (p < 0.001), with the VR group showing the greatest improvement, followed by the regular training group.
6.	da Cruz Netto et al. [29]	2020	To assess the effectiveness of a VR environment in improving sequential actions of daily routine memory.	Memorization of daily routine	Pre- and post-comparison within the groups	Control group (mean difference pre-and post) = 0.1332, whereas, VR group = 0.7334. The VR group showed significantly better daily task memorization than the control group (p < 0.0001), with an 81.82% higher improvement.
7.	Højberg et al. [30]	2023	To explore how new motor skills were acquired and retained in young adults with DS.	Participants completed seven blocks (10.6 min) of a VATT. Motor performance was evaluated at baseline, immediately after practice, and after seven days to assess online and offline learning effects.	Pre- and post-comparison within the groups and in between the groups.	The TD group outperformed the DS group across all blocks (~30 points better). Both groups showed similar online improvement from baseline to immediate retention. However, in the offline effect, the DS group maintained their performance at the 7-day retention, while the TD group experienced a decline (TD = 5.8 ± 1.5, P < 0.001; DS = 7.9 ± 1.7, P < 0.001).

Physical activity and functional performance

Rosa et al. (2023) used the VR game MoveHero [25]. The patients were involved for a total of 11 sessions. The outcomes of Rate of Perceived Exertion (RPE) and heart rate (HR) were measured at rest and during gameplay. The study reported that VR offers a promising approach to increasing PA and reducing sedentary behavior, as evidenced by improved heart rate, perceived effort, and motor performance with practice. Hence, VR can enhance rehabilitation by improving fitness, reducing sedentary behavior, and preserving functionality in DS patients.

Static and dynamic balance

Ghouri et al. (2024) involved children (intelligent quotient (IQ) of 50-70%) who could stand, walk independently, and follow instructions [26]. Whereas those with autism, sensory impairments, epilepsy, muscular dystrophies, or traumatic brain injury were excluded. Balance and motor control were assessed using the pediatric balance scale (PBS) and Rhomberg tests. Both groups showed significant within-group improvements in PBS scores, but no between-group differences were found in PBS or Rhomberg test outcomes. The study concluded that both groups improved static and dynamic balance significantly, with a greater effect observed in static balance in the VR group. Similarly, Yunus et al. (2024) included the VR SenMor group that received therapy twice weekly for four weeks, while the control group had no intervention [27]. The outcomes measured were the PBS and timed-up and go test (TUG) test pre- and post-treatment for balance. The study reported that VR SenMor therapy may improve static and dynamic balance in DS patients.

Memory and learning behavior

Michalski et al. (2022) evaluated the feasibility and benefits of VR for learning [17]. Both VR (P<0.001) and conventional (P=0.002) drawing significantly improved behavior, mood, and attention in a learning setting, with no significant difference between interventions (P=0.53). Hence, the findings support VR's feasibility for learning in DS patients. Similarly, da Cruz Netto et al. (2020) assessed the effectiveness of a VR environment in improving sequential actions of daily routine memory [29]. The study concluded that the VR environment's playful activities engaged children, encouraging enjoyment, hypothesis testing, and reflection on daily task sequences. 

Acquisition of motor skills and their retention

Højberg et al. (2023) explored how new motor skills were acquired and retained in young adults with DS [30]. Participants completed seven blocks (10.6 min) of a visuomotor accuracy tracking task (VATT). Motor performance was evaluated at baseline, immediately after practice, and after seven days to assess online and offline learning effects. Hence, the study concluded that adults with DS exhibit lower visuomotor pinch force accuracy than typically developed (TD) individuals but show comparable online improvements with practice. They also demonstrate offline consolidation, leading to significant skill retention.

Independent functional skills

The study by Gaber SA (2024) utilized an interactive three-dimensional (3D) VR training program [28]. The program, based on the “Addie Model”, aimed to enhance independence skills and was assessed using the ISS. The program consisted of three stages, a four-session introductory phase focused on familiarization and engagement. The training phase (38 sessions) covered essential life skills: eating (Sessions 5-14), drinking (Sessions 15-19), dressing and undressing (Sessions 20-31), and personal hygiene and self-care (Sessions 32-42). The final evaluation stage (8 sessions) assessed skill acquisition and performance. Hence, the study concluded that a VR-based training program should be integrated into educational programs for children with moderate DS to enhance independence and skill development.

Discussion

This SR analyzed a total of seven RCTs that evaluated the effectiveness of VR-based interventions in improving motor skills, balance, and cognitive functions in DS patients, with studies reporting high participant engagement and adherence. The findings reported in the present review confirm the effectiveness of VR-based intervention in improving physical activity levels and functional performance, enhancing static and dynamic balance, acquiring and retaining new motor skills, improving learning behavior and memory, and enhancing independent functional skills [17,25-30]. Additionally, the ability of VR to provide a game-based interactive environment appears to enhance motivation, which is often a challenge in traditional rehabilitation settings. Compared to conventional rehabilitation methods, VR interventions allow real-time feedback, immersive experiences, and task-specific motor learning, which can contribute to functional improvements [12].

Rosa et al. (2023) highlighted VR as a promising tool for promoting physical activity and reducing sedentary behavior in DS patients, as evidenced by increased heart rate and perceived effort in both groups. Additionally, the study reported significant improvements in motor performance with repeated practice [25]. Moreover, Ghouri et al. (2024) reported significant improvements in balance (static and dynamic) across all groups, with the VR intervention demonstrating a greater impact on static balance in patients with DS [26]. Furthermore, Yunus et al. (2024) concluded that VR SenMor therapy holds the potential for improving balance (static and dynamic) in children with clinical DS [27]. Similarly, VR-based interventions positively impacted postural control and motor skills, as reported by Gómez Álvarez et al. (2018) [10], supporting the notion that technology-assisted therapies provide engaging and effective rehabilitation options for individuals with DS [23]. The feasibility of telerehabilitation approaches was explored by Ptomey et al. (2018), who demonstrated that video-based exercise sessions were effective in increasing moderate-to-vigorous physical activity levels among DS patients [34]. Moreover, Silva et al. (2017) reported that Wii-based exercise effectively enhances mobility, motor skills, and physical fitness in DS patients, particularly lower limb strength and aerobic capacity. Exergames like Wii Fit offer an engaging alternative to reduce sedentary behavior, promote regular activity, and lower cardiovascular risk [11]. Hence, these results were consistent with previous findings that suggest digital health interventions can enhance accessibility to physical activity programs, especially for populations with mobility limitations [35].

The current review also explored the effectiveness of VR exposure in learning settings for DS patients [25] and integration into diverse training and educational programs to foster independence and skill development [26]. However, some discrepancies exist in the literature. While Ghouri et al. (2024) observed significant within-group improvements in balance outcomes among DS individuals following VR training, their between-group analysis revealed no statistically significant differences compared to conventional therapy [26]. Additionally, adults with DS exhibit lower visuomotor pinch force accuracy than TD individuals but show comparable online improvements with practice. They also demonstrate offline consolidation, leading to significant skill retention [30]. This finding is consistent with earlier studies by Michalski et al. (2022), which suggested that while VR interventions provide promising outcomes, their effects may not always surpass traditional rehabilitation methods, particularly in short-term interventions [17].

Furthermore, da Cruz Netto et al. (2020) reported that VR training enhanced memory and daily functional skills in children with DS, indicating cognitive benefits beyond motor improvements [29]. These findings reinforce the broader applicability of VR beyond physical rehabilitation, aligning with the work of Nugent et al. (2018), who suggested that VR-based educational tools could support self-care training and cognitive skill development in DS individuals [36]. The study also highlighted certain disparities that involved healthcare access and transition planning as in comparison to patients with special healthcare needs, adolescents with DS were less likely to receive healthcare transition planning [36], reflecting a broader issue of insufficient provider awareness and support for transition services. Similarly, Ahlström et al. (2020) reported that older adults with DS had fewer planned healthcare visits despite having a high prevalence of preventable conditions, reinforcing the need for improved adherence to medical guidelines to prevent unplanned hospitalizations [37].

Strengths and limitations

The review provides a broad perspective on the efficacy of VR in DS rehabilitation. The included studies assessed multiple domains, including motor performance, postural control, functional independence, and cognitive abilities. Hence, technology-assisted interventions are promising alternatives for engaging individuals with DS in structured therapy. Moreover, studies included in this review adhered to standardized outcome assessments such as the Timed Up and Go (TUG) test, Pediatric Balance Scale (PBS), and task-based VR assessments, ensuring the reliability of findings. However, the limitation of the review involves the heterogeneity of included studies as the reviewed studies employed different VR platforms, intervention durations, and training protocols. Additionally, a restricted quantity of RCTs, a small sample size, and limited longitudinal data to determine the long-term sustainability of improvements induced by VR were the limitations observed. Moreover, a meta-analysis was not feasible due to variability in study designs and outcome measures, thus limiting the ability to derive pooled effect estimates and make stronger statistical inferences about the efficacy of therapeutic interventions. Furthermore, only three databases were included for the present review, and only studies present in full-text English were included, which may limit knowledge access across various databases.

Recommendations and future directions

The recommended future directions consist of the conduction of large-scale, multi-center RCTs with standardized intervention protocols, outcome measures, and comparison with conventional interventions, implementation of long-term follow-ups to assess the sustainability of therapeutic benefits, integration of objective assessments and biomarkers to enhance the accuracy of reported outcomes, and conduction of meta-analyses to quantify the overall effectiveness of therapeutic interventions. Additionally, for future review articles, more databases for screening and selection of the relevant studies can be incorporated as the present review involved only three databases. Moreover, studies presented in other languages including English can be considered for the future scope. By addressing these gaps, future studies can contribute to more robust and clinically applicable recommendations for optimizing VR-based therapeutic intervention in individuals with DS.

## Conclusions

This systematic review analyzed the impact of VR-based interventions in enhancing significant motor and cognitive functions in DS patients. The reviewed studies suggest that VR can be an effective tool for improving postural balance, motor coordination, and engagement in rehabilitation. However, variations in study methodologies and sample characteristics indicate the need for further well-designed trials to establish standardized protocols and assess long-term outcomes. Given the growing accessibility and advancements in VR technology, its integration into DS rehabilitation programs may provide an engaging and effective alternative to conventional therapies. Future research should focus on optimizing VR intervention strategies, exploring personalized approaches, and evaluating cost-effectiveness to facilitate widespread clinical implementation. Hence, by acknowledging existing knowledge gaps and directing future research efforts, evidence-based practices can be refined, consequently leading to the improvement of the lives of DS patients worldwide.
